# A systemic review and meta-analysis of the clinical efficacy and safety of total glucosides of peony combined with methotrexate in rheumatoid arthritis

**DOI:** 10.1007/s10067-017-3770-y

**Published:** 2017-07-27

**Authors:** Zhi-tao Feng, Juan Xu, Guo-chao He, San-jin Cai, Juan Li, Zhi-gang Mei

**Affiliations:** 1 Third-Grade Pharmacological Laboratory on Chinese Medicine Approved by State Administration of Traditional Chinese Medicine, Medical College of China Three Gorges University, Yichang, Hubei 443002 China; 2 Shenzhen Institute of Geriatrics, Shenzhen, Guangdong 518020 China; 3 Department of Rheumatology, Nanfang Hospital, Southern Medical University, Guangzhou, Guangdong 510515 China; 4 Department of Traditional Chinese Internal Medicine, School of Traditional Chinese Medicine, Southern Medical University, Guangzhou, Guangdong 510515 China; 5 Department of Orthopedic Surgery, Hunan Provincial Hospital of Traditional Chinese Medicine, Zhuzhou, Hunan 412008 China

**Keywords:** Meta-analysis, Methotrexate, Randomized controlled trial, Rheumatoid arthritis, Systematic review, Total glucosides of peony, Traditional Chinese medicine

## Abstract

To assess the efficacy and safety of the combination of total glucoside of peony (TGP) and methotrexate (MTX) for the treatment of rheumatoid arthritis (RA). Randomized controlled trial (RCT) data on the traditional Chinese active component TGP combined with MTX vs. MTX alone for the treatment of RA was collected by searching the Pubmed, Embase, Cochrane Library, CNKI, VIP Journals database, and Wanfang database up to February 2017. Study selection, data extraction, data synthesis, and data analyses were performed according to the Cochrane standards. A total of eight RCTs involving 522 participants were included in this meta-analysis. Compared with MTX alone, the use of TGP combined with MTX exhibited better therapeutic effects for the treatment of RA (*P* = 0.004). In addition, TGP combined with MTX caused a more significant decrease in erythrocyte sedimentation rate (ESR) (*P* < 0.0001) and swollen joint count (SJC) (*P* < 0.00001). However, no significant differences were found in C-reactive protein (CRP) (*P* = 0.19), duration of morning stiffness (DMS) (*P* = 0.32), or tender joint count (TJC) (*P* = 0.23) between the two groups. In addition, adverse events were more frequently reported in the MTX monotherapy group than in the TGP and MTX combination group (*P* = 0.0007). Our study demonstrates that TGP combined with MTX is more effective than MTX alone for the treatment of RA. Nevertheless, the adverse effects of the combination of TGP and MTX need to be further assessed. Due to the poor methodological quality of included trials, well-designed, multi-center, and large-scale RCTs are necessary to draw a more definitive conclusion.

## Introduction

Rheumatoid arthritis (RA) is one of the most common autoimmune diseases in the world and is characterized by synovial inflammation, hyperplasia, autoantibody production, and cartilage and bone destruction [1, 2]. RA can cause a loss of joint function, reduce quality of life, and enhance mortality. Approximately 1% of the worldwide population is affected by RA [3]. Its pathogenesis is not fully understood, rendering efforts towards preventative treatment ineffective [4]. The current treatment strategies for RA include nonsteroidal anti-inflammatory drugs (NSAIDs), corticosteroids, disease-modifying anti-rheumatic drugs (DMARDs), biologic response modifiers (biologicals), and traditional Chinese medicine (TCM) [5–8]. The recommended treatment model begins with DMARDs after RA diagnosis [5]. The treatment generally begins with methotrexate (MTX), which acts as an “anchor drug” [9]. TCM and TCM preparations have unique advantages for the treatment of RA, such as overall adjustment, multi-level, multiple targets, and fewer side effects [10, 11]. In recent years, the potential and positive effects of TCM have increasingly attracted public interest for the treatment of autoimmune diseases.

Total glucoside of peony (TGP) is a biologically active compound extracted from roots of *Paeonia lactiflora* Pall [12] and is a traditional Chinese medicine that has been used to treat RA for centuries. Pharmacological studies have indicated that TGP has anti-inflammatory [13–16], immune-regulatory [17, 18], and analgesic effects [19, 20]. In addition, TGP was shown to reduce joint pain and swelling and inhibit joint damage [21, 22]. Base on these potential properties, TGP (trade name: pafulin) is widely used for the treatment of RA in China. Currently, several clinical studies have demonstrated a significant improvement of RA symptoms and the prevention of RA progression afforded by TGP combined with MTX compared to MTX monotherapy. However, the results of these studies are inconsistent, and very little is known about the side effects of the combined therapy. Therefore, we conducted this meta-analysis of randomized controlled trials (RCT), in order to systematically evaluate the efficacy and safety of TGP combined with MTX vs. MTX monotherapy for the treatment of RA. We hypothesized that the results of this study could provide evidence for the superiority of treating RA with TGP plus MTX.

## Materials and methods

### Search strategy

The Pubmed, Embase, Cochrane Library, China National Knowledge Infrastructure (CNKI), VIP Journals database, and Wanfang databases were searched to collect published studies for the present meta-analysis. All of these databases were searched for relevant data from their inception to the latest issue (February 2017). A number of words including “rheumatoid arthritis,” “RA,” “total glucoside of peony,” “Pafulin,” “TGP,” “methotrexate,” and “MTX” were utilized as both medical subject heading (MeSH) terms and text words to identify all articles having reported on the combination of TGP and MTX for the treatment of RA. A manual search of references from original research or review articles was performed to identify additional studies. No language or time restrictions were applied in the search.

### Study selection

Trials were considered to be eligible for inclusion if they met all of the following criteria: (i) all patients fulfilled the 1987 revised American College of Rheumatology criteria for the disease [23], (ii) the RCTs, the evaluated efficacy, and safety of TGP combined with MTX for RA patients, (iii) outcomes included at least one of the following: therapeutic effects (TEs), erythrocyte sedimentation rate (ESR), rheumatoid factor (RF), C-reactive protein (CRP), duration of morning stiffness (DMS), swollen joint count (SJC), tender joint count (TJC), and adverse events (AE). There were no restrictions regarding gender, age, severity or duration of RA.

### Data extraction

The relevant data were extracted by two independent reviewers (Zhitao Feng and Juan Xu), including study design, randomization, diagnostic criteria, the first author’s name, publication year, sample size, treatment duration, dose, outcomes, and AEs. Disagreements were resolved by consensus or were arbitrated by a third investigator (Juan Li or Zhigang Mei).

### Statistical analysis

Review Manager 5.3 software (Cochrane Collaboration, Oxford, UK) was used to analyze the data. The quality of each included study was assessed according to the Cochrane Handbook for Systematic Reviews of Interventions and Jadad scoring [24]. Odds ratios (OR) with 95% confidence intervals (CIs) were calculated for dichotomous data. If continuous data were available, the mean difference (MD) with 95% CIs was calculated. A fixed-effect model was employed if there was no statistical heterogeneity among studies; otherwise, the random-effect model was used [25]. Cochrane’s chi-square test was used to assess heterogeneity, and Higgins *I* ^2^ measures the degree of inconsistency between studies whether the percentage total variation across studies is due to heterogeneity rather than chance [8, 26]. Publication bias was detected by the use of funnel plots and the Egger regression asymmetry test (Stata software, version 12.0).

## Results

### Study selection and characteristics

**Fig. 1:**
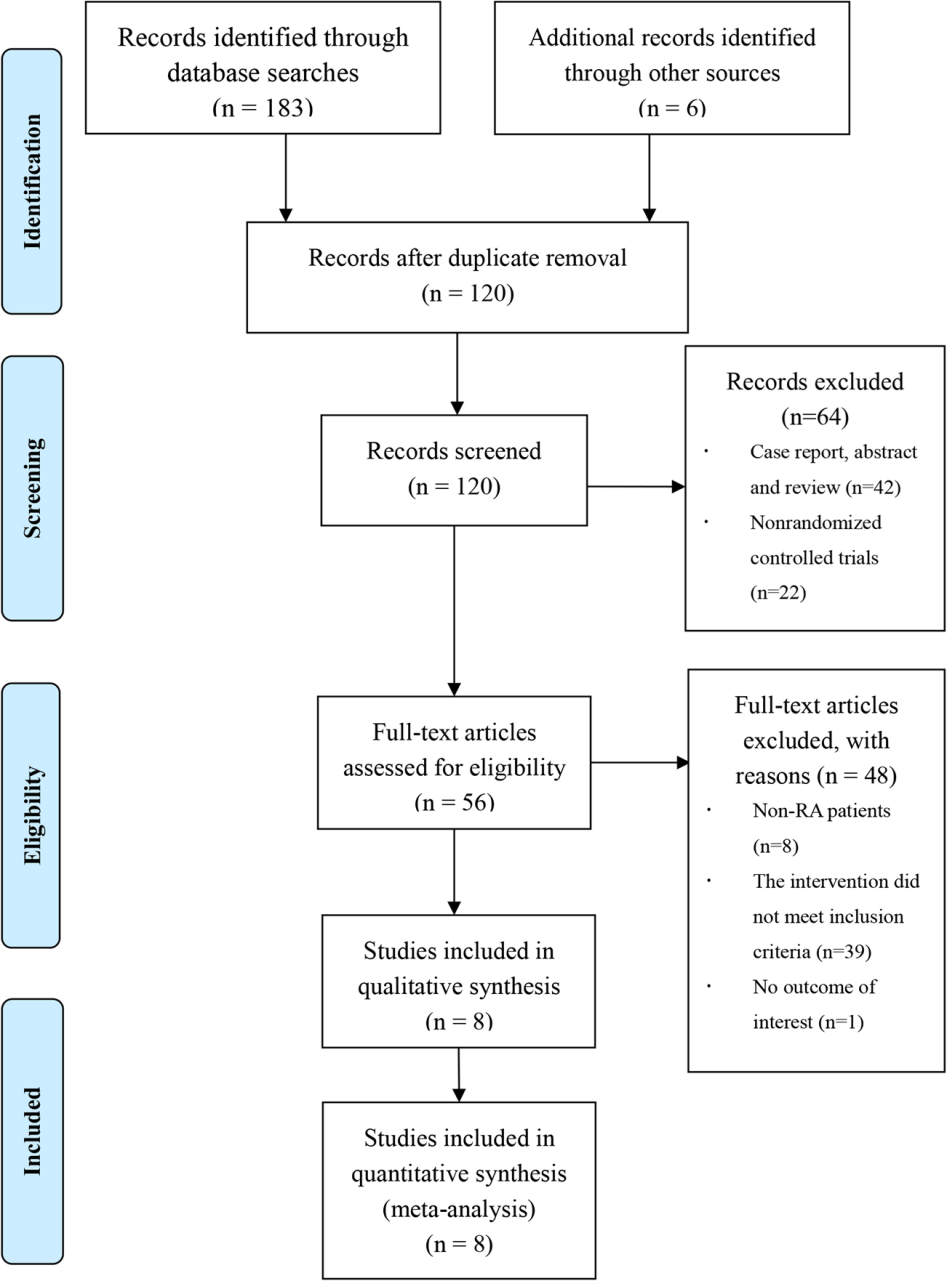
Flow diagram of study selection process

Employing the abovementioned search strategy, we retrieved 189 potentially relevant articles. After removal of duplicates, 120 studies were screened. The content review limited the relevant papers to eight studies that met the inclusion criteria for the meta-analysis [27–34]. The general procedure for study selection is shown in Fig. 1.

A total of eight trials including 265 RA cases and 257 controls that met our inclusion criteria were included in the present study. The participant numbers in the individual studies varied from 46 to 90. The TGP doses ranged between 0.9 and 1.8 g/day but most were 1.8 g/day. The dose of MTX in the combination therapy and monotherapy groups ranged between 7.5 and 15 mg/week. The duration of the interventions in the included studies varied from 12 to 24 weeks and 6 studies described TEs evaluated on the basis of four classes of outcomes such as “cure,” “significant effective,” “effective,” and “ineffective” [27–29, 31, 32, 34]. Four of the trials discussed the AEs in detail [27, 29, 30, 34]. In addition, five mentioned the ESR [27–29, 33, 34]; two mentioned the CRP [27, 33]; one mentioned the RF [33]; four mentioned the DMS [27–29, 34]; three mentioned the TJC [28, 29, 34]; and three analyzed SJC [28, 29, 34]. The characteristics of the included RCTs are summarized in Table 1.

**Table 1:** Characteristics of the eight trials included in the meta-analysis

Author	Participants		Age		Interventions		Duration	Outcomes
	E (M/F)	C (M/F)	E	C	E	C		
Zhang 2010 [27]	42 (13/29)	42 (12/30)	41.3 ± 13.4	40.9 ± 12.3	TGP 0.3 g tid, MTX 10 mg qw	MTX 10 mg qw	24 weeks	TE, ESR, CRP, DMS, AE
Ma 2010 [28]	30	30	NA	NA	TGP 0.6 g bid, MTX 7.5~15 mg qw	MTX 7.5~15 mg qw	24 weeks	TE, ESR, DMS, TJC, SJC
Shang et al. 2009 [29]	31 (11/20)	28 (9/19)	40 ± 6	39 ± 6	TGP 0.6 g tid, MTX 7.5 mg qw	MTX 7.5 mg qw	12 weeks	TE, ESR, DMS, TJC, SJC, AE
Zhu 2009 [30]	23 (6/17)	23 (7/16))	46 ± 12	47 ± 11	TGP 0.6 g tid, MTX 7.5 mg qw	MTX 15 mg qw	24 weeks	TE, AE
Liu et al.2007 [31]	46	44	NA	NA	TGP 0.6 g tid, MTX 7.5~10 mg qw	MTX 7.5~10 mg qw	24 weeks	TE, AE
Wang et al.2007 [32]	32	30	NA	NA	TGP 0.6 g tid, MTX 7.5 mg qw	MTX 7.5 mg qw	12 weeks	TE, AE
Yin et al.2007 [33]	30	30	NA	NA	TGP 0.6 g bid, MTX 10 mg qw	MTX 10 mg qw	12 weeks	ESR, CRP, RF, AE
Du et al.2005 [34]	31	30	NA	NA	TGP 0.6 g tid, MTX 15 mg qw	MTX 15 mg qw	12 weeks	TE, ESR, DMS, TJC, SJC, AE

*TGP* total glucosides of peony, *MTX* methotrexate, *TE* therapeutic effect, *ESR* erythrocyte sedimentation rate, *CRP* C-reactive protein, *RF* rheumatoid factor, *DMS* duration of morning stiffness, *TJC* tender joint count, *SJC* swollen joint count, *AE* adverse event, *E* experiment group, *C* control group, *M* male, *F* female, *NA* not available

### Risk of bias assessment

**Fig. 2:**
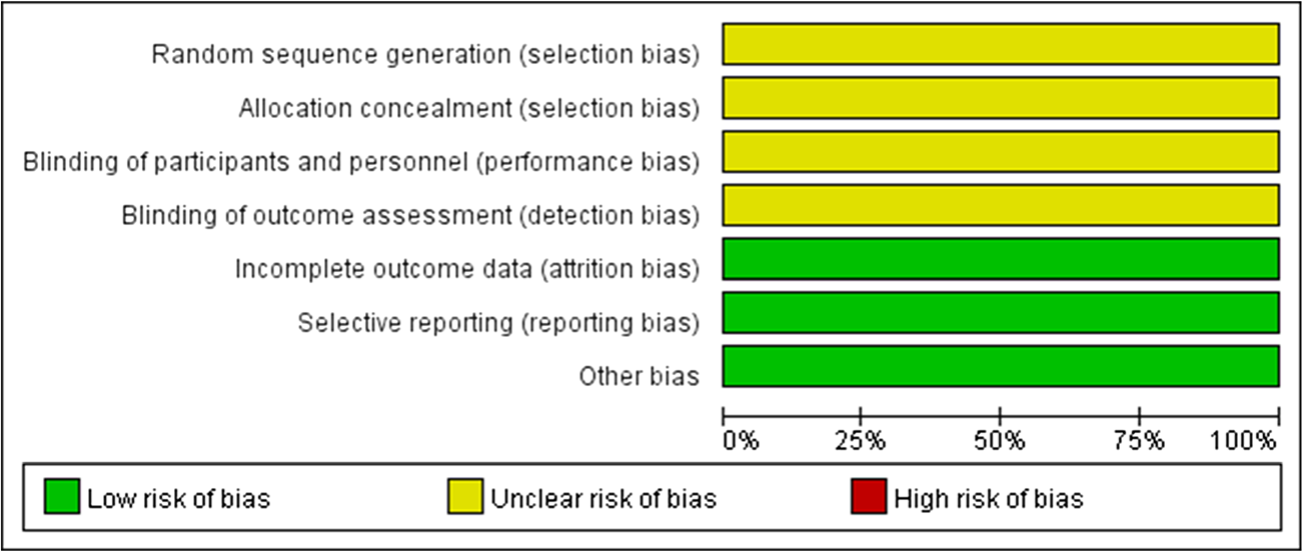
Risk of bias assessment

Most included RCTs exhibited poor methodological quality according to the criteria shown in Fig. 2. All the trials described the randomization procedure, but none reported allocation concealment. Insufficient information was available for assessment of the allocation method. In addition, no study described the blinding of participants or dropout information.

### The TEs of TGP combined with MTX vs. MTX alone

**Fig. 3:**
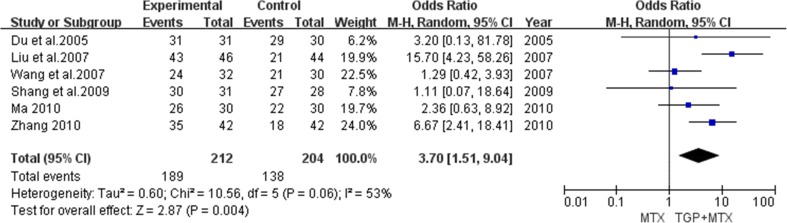
Meta-analysis of the TEs of TGP combined with MTX vs. MTX alone. *TE* therapeutic effect, *TGP* total glucosides of peony, *MTX* methotrexate

The TEs of TGP combined with MTX vs. MTX alone were compared in six RCTs including of a total of 416 patients. As shown in Fig. 3, the pooled results from these trials showed a significant difference in the TEs between TGP combined with MTX and those of MTX alone (*P* = 0.004; OR = 3.70; 95% CI 1.51 to 9.04) based on the random-effect model (*I* ^2^ = 53%, *P* = 0.06).

### ESR (mm/h), CRP (mg/L), DMS, TJC, and SJC

**Fig. 4:**
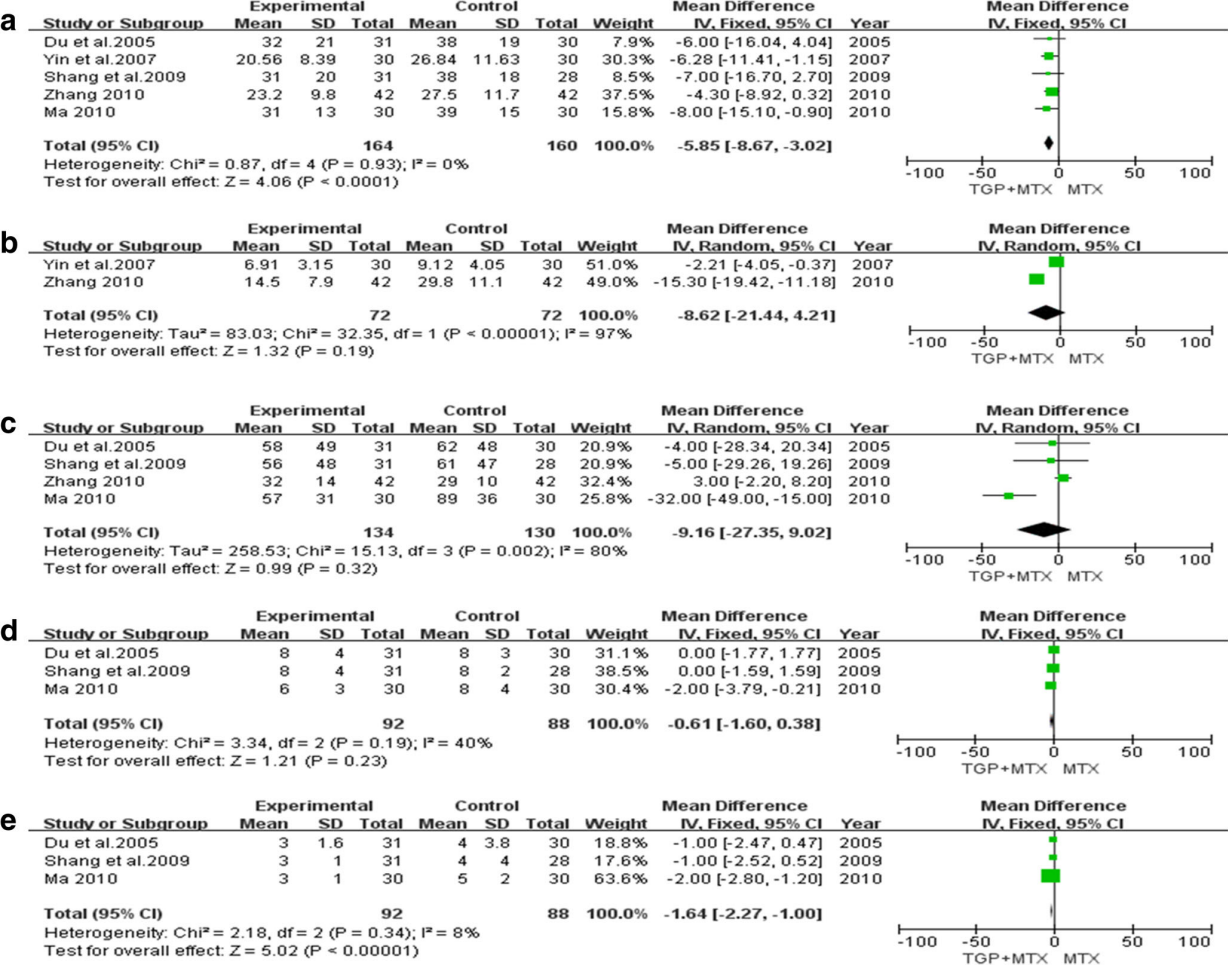
Meta-analysis of the effects of the combination of TGP and MTX vs. MTX alone on serum levels of ESR, CRP, DMS, TJC, and SJC. **a** ESR. **b** CRP. **c** DMS. **d** TJC. **e** SJC. *TGP* total glucosides of peony, *MTX* methotrexate, *ESR* erythrocyte sedimentation rate, *CRP* C-reactive protein, *DMS* duration of morning stiffness, *TJC* tender joint count, *SJC* swollen joint count

Five trials reported the effects of the combination of TGP and MTX vs. MTX alone on ESR serum levels, and a fixed-effect model was used to analyze the data (*I* ^2^ = 0%, *P =* 0.93). The results revealed obvious differences in ESR serum levels between TGP combined with MTX and those of MTX alone (*P* < 0.0001; MD = −5.85, 95% CI = −8.67 to −3.02), as shown in Fig. 4a.

Two trials reported the effects of the combination of TGP and MTX vs. MTX alone on CRP serum levels, and a random-effect model was used to analyze the data (*I* ^2^ = 97%, *P* < 0.00001). The results failed to find any significant differences in CRP serum levels between the two groups, however (*P* = 0.19; MD = −8.62, 95% CI = −21.44 to 4.21), as shown in Fig. 4b.

Four trials reported the effects of the combination of TGP and MTX vs. MTX alone on DMS, and a random-effect model was used to analyze the data (*I* ^2^ = 80%, *P* = 0.002). The results displayed no significant differences in the DMS between the two groups (*P* = 0.32; MD = −9.16, 95% CI = −27.35 to 9.02), as shown in Fig. 4c.

Three trials reported the effects of the combination of TGP and MTX vs. MTX alone on TJC, and a fixed-effect model was used to analyze the data (*I* ^2^ = 40%, *P* = 0.19). The results failed to find any significant differences in TJC between TGP combined with MTX and that of MTX alone (*P* = 0.23; MD = −0.61, 95% CI = −1.60 to 0.38), as shown in Fig. 4d.

Three trials reported the effects of the combination of TGP and MTX vs. MTX alone on SJC, and a fixed-effect model was used to analyze the data (*I* ^2^ = 8%, *P* = 0.34). The results displayed remarkable differences in SJC between TGP combined with MTX and MTX alone (*P* < 0.00001; MD = −1.64, 95% CI = −2.27 to −1.00), as shown in Fig. 4e.

### AEs

**Fig. 5:**
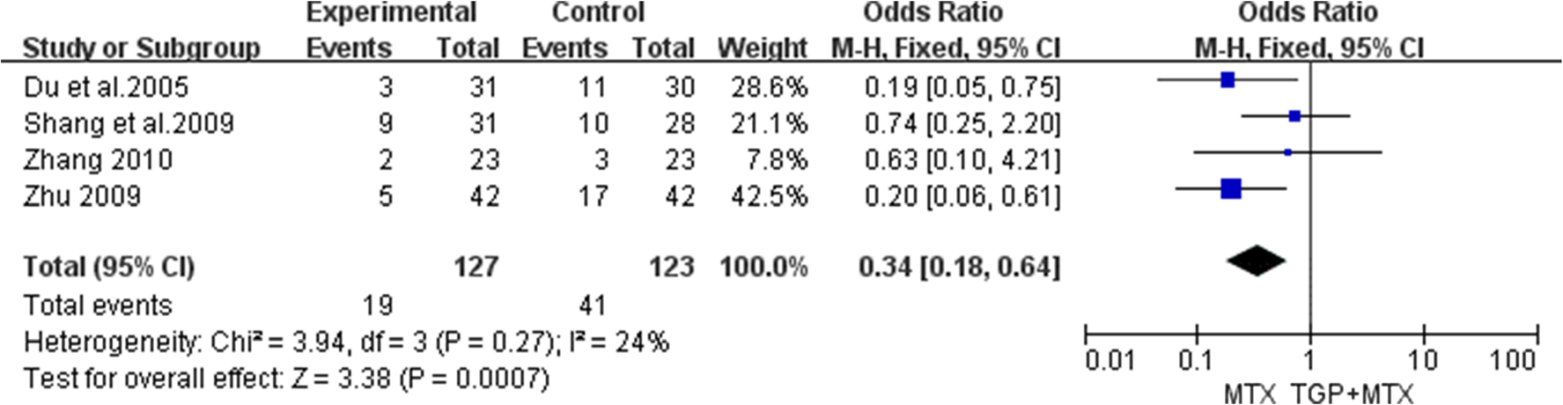
Meta-analysis of AEs of TGP combined with MTX vs. MTX only for the treatment of RA. *TGP* total glucosides of peony, *MTX* methotrexate, *AE* adverse event

Seven studies reported AEs caused by TGP combined with MTX or MTX alone. AEs were not mentioned in one of the studies [28], but four out of seven trials [27, 29, 30, 34] recorded AE cases in two groups. No heterogeneity was identified among the studies (*P* = 0.27 and *I* ^2^ = 24%) based on the fixed-effect model. As shown in Fig. 5, the results of our meta-analysis revealed a significantly higher rate of AEs induced by MTX alone than by TGP combined with MTX (*P* = 0.0007; OR = 0.34; 95% CI 0.18 to 0.64). Four trials [27, 30, 33, 34] reported mild abnormal liver function, and five trials [27, 29, 30, 33, 34] recorded mild to moderate gastrointestinal events. These AEs mentioned above were relieved or disappeared after symptomatic treatment. No serious AEs were recorded.

### Publication bias

**Fig. 6:**
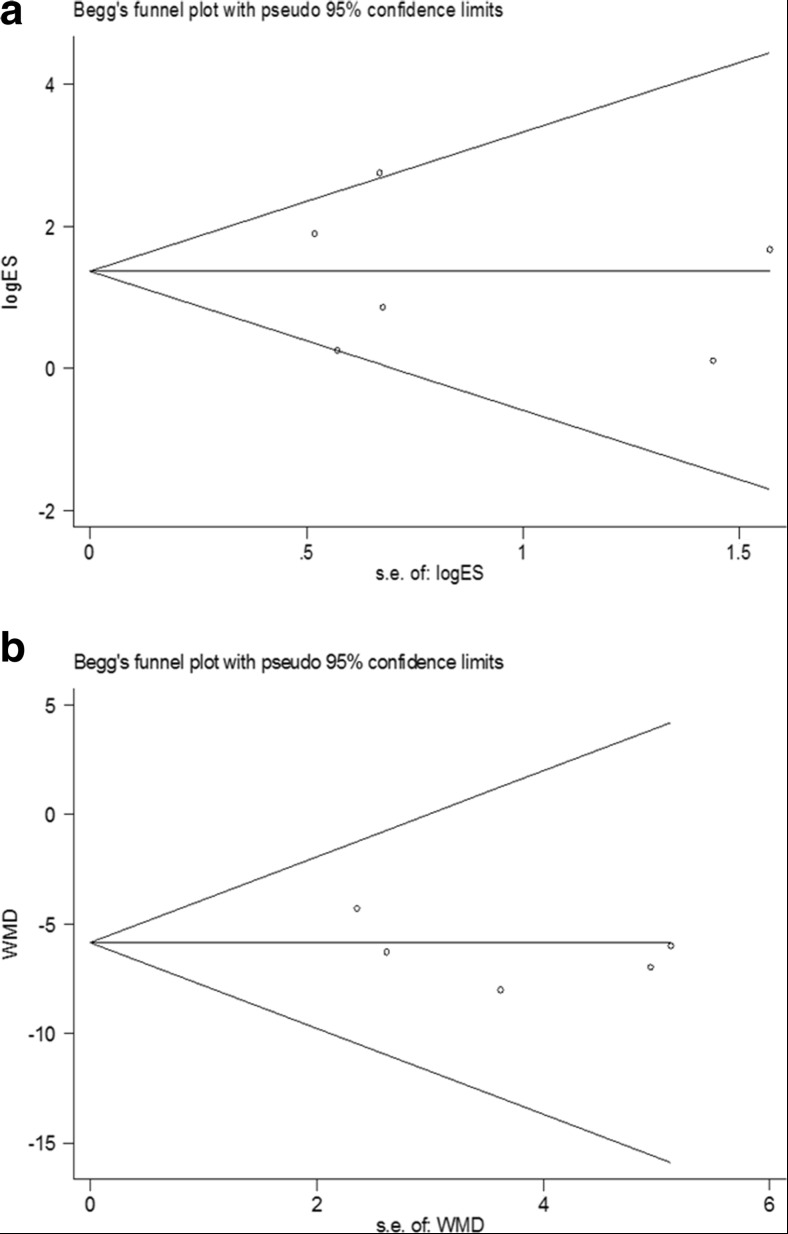
The Begg funnel plots for publication bias in the included trials. **a** TE. **b** ESR. *TE* therapeutic effect, *ESR* erythrocyte sedimentation rate

Begg’s publication bias plots showed that there were no significant publication biases when five or more studies were included (Fig. 6a, b). The results of the Egger test also showed that there was no significant publication bias (TEs, *P* = 0.825; ESR, *P* = 0.306).

## Discussion

This article provides a PRISMA-compliant meta-analysis and systematic review of the efficacy and safety of TGP combined with MTX in the treatment of RA. We previously investigated the efficacy and safety of the combination of TGP and leflunomide (LEF) on RA, and the results showed that TGP combined with LEF was more effective and safer than LEF alone for the treatment of RA [35]. In the present study, we included eight RCTs recording the TEs of TGP combined with MTX vs. those of MTX alone for the treatment of RA. A total of 265 patients in the treatment group (TGP combined with MTX) and 257 in the control group (MTX alone) were assessed. The pooled data suggests that TGP combined with MTX produces better therapeutic effects than MTX treatment alone. As shown in Fig. 4, the oral administration of TGP combined with MTX induced better effects on ESR and SJC than did MTX alone. Moreover, the pooled results indicate a significantly lower rate of AEs induced by TGP combined with MTX than was found in MTX alone.

Some related findings might help to explain the potential TEs of TGP combined with MTX in the treatment of RA. Previous pharmacological studies have concluded that TGP has crucial anti-inflammation, immunosuppression, and analgesic properties, as well as inhibiting bone destruction. TGP has been reported to possess anti-inflammatory effects by regulating pro-inflammatory mediators and suppressing the proliferation of fibroblast-like synoviocytes in collagen-induced arthritis (CIA) or adjuvant arthritis (AA) model [13–16]. In addition, studies have shown that TGP inhibits both the maturation and function of dendritic cells (DCs) in vivo, which in turn results in reducing immune-mediated inflammation in vivo [17, 18]. Moreover, paeoniflorin, the major active ingredient of TGP [12], has a prominent analgesic effect on visceral pain caused by colorectal distension in the rats with visceral hyperalgesia induced by neonatal maternal separation [19, 20]. It is worth noting that TGP exerts a significant inhibiting effect on joint destruction in CIA [21] or antigen-induced arthritis (AIA) models [22].

Another, MTX is a folic acid antagonist and inhibits the synthesis of DNA, RNA, and proteins by binding to dihydrofolate reductase. Currently, MTX is the most widely used as a DMARD for the treatment of both early and established RA and has been named an “anchor agent” for combination therapy with other DMARDs and biological agents [9, 36]. Several mechanisms have been proposed to explain the effects of MTX in RA, including the antagonism of folate-dependent processes, stimulation of adenosine signaling, generation of reactive oxygen species (ROS), downregulation of adhesion-molecule expression, regulation of cytokine profiles, and downregulation of eicosanoids and matrix metalloproteinases (MMPs) [36]. In the present study, we compared the TEs of the combination of TGP and MTX to those of MTX alone for the treatment of RA, and the results indicated that the combination group exhibited efficacy than the monotherapy group, suggesting that TGP could be used as a type of “herbal DMARD” for complementary RA therapeutics.

Nonetheless, our meta-analysis has several limitations which should be taken into consideration when interpreting our results. First, all of the included studies were conducted in Chinese populations, which presented a high risk of selection bias. Second, the quality scoring system of the included trials was not well established. The randomization procedures were not described in all eight studies. In addition, the general lack of information on allocation concealment, blinding, dropouts, and intention to treat may conceal potential selection or detection biases. Third, publication bias is possible because all of the studies were published in Chinese, although we did conduct comprehensive searches in both English and Chinese publication databases for relevant articles. Fourth, the outcome measures were inadequate, and only one study described the clinical outcomes defined by the American College of Rheumatology (ACR) criteria [30]. Besides that, differences in the quality of trials, intervention methods, doses, and treatment duration were responsible for the heterogeneity. Based on the above, we suggest that future researchers focus on the following criteria. RCTs of the efficacy and safety of TGP combined MTX in treating RA need larger sample sizes, multiple centers, and longer follow-up times. And RCTs should describe their randomization, allocation concealment, blinding, and other information. Moreover, researchers should formulate strict inclusion and exclusion criteria and standardize both outcome evaluation indicators and safety analysis. Consequently, the conclusions of this study should be carefully interpreted.

## Conclusions

In summary, the results of the present study suggest that TGP combined with MTX is more effective than MTX alone for the treatment of RA. Therefore, TGP can be used as a complementary DMARD. In addition, the AEs should be further evaluated. Given that the quality of included trials was moderate to low, well-designed, multi-center, and large-scale RCTs are necessary to further confirm our results.
